# 应用Clavien-Dindo分级系统对肺癌患者术后并发症分级及危险因素分析

**DOI:** 10.3779/j.issn.1009-3419.2017.04.07

**Published:** 2017-04-20

**Authors:** 鹏飞 李, 玉田 赖, 坤 周, 国卫 车

**Affiliations:** 610041 成都，四川大学华西医院胸外科 Department of Thoracic Surgery, West China Hospital, Sichuan University, Chengdu 610041, China

**Keywords:** 肺肿瘤, 术后并发症, Clavien-Dindo分级, 危险因素, Lung neoplasms, Postoperative complications, Clavien-Dindo classification, Risk factors

## Abstract

**背景与目的:**

术后并发症是肺切除术后患者死亡的重要原因。在本研究中，我们应用Clavien-Dindo并发症分级系统对肺癌术后并发症按照严重程度进行分级，并分析术后并发症的发生率，探讨不同分级术后并发症的危险因素。

**方法:**

回顾性分析2013年6月-2014年12月四川大学华西医院胸外科966例行肺叶切除术的肺癌患者，依据术后30 d内是否发生并发症将此966例患者分为并发症组与无并发症组；同时根据Clavien-Dindo分级系统将并发症分为4级，并针对不同分级的并发症进行危险因素分析。

**结果:**

966例患者中，并发症组占15.0%（145/966），发生总数380次；依据Clavien-Dindo分级系统将此380次并发症进行分级，其中Ⅰ级、Ⅱ级、Ⅲ级、Ⅳ级及以上分别占6.8%、75.3%、15.0%和2.9%。*Logistic*回归分析结果显示术前第1秒用力呼气容积（forded expiratory volume in one second, FEV_1_）、肺一氧化碳弥散量（diffusion capacity for carbon monoxide of the lung single breath, DLco SB）及术前合并慢性阻塞性肺疾病（chronic obstructive pulmonary disease, COPD）是术后并发症的独立危险因素；其中术前FEV_1_是Ⅰ级、Ⅱ级、Ⅲ级及以上并发症的独立危险因素。

**结论:**

在Clavien-Dindo分级系统下，Ⅱ级并发症在术后30天内最常见；FEV_1_与术后并发症的发生密切相关，可作为评估术后并发症发生风险的可靠指标之一。

## Abstract (en)

**Background and objective:**

Postoperative complications (PCs) are contributing factors to patient mortality following lung resection. In this retrospective study, the Clavien-Dindo classification was used to analyze the current incidence of PCs and identify the risk factors of different grades of PCs.

**Methods:**

A total of 966 lung cancer patients who underwent lobectomy in our department between June 2013 and December 2014 were retrospectively enrolled. Patients were divided into two groups depending on the occurrence of PCs. The PCs were classified into four grades through the Clavien-Dindo classification, and the related risk factors of different grades were evaluated.

**Results:**

Approximately 15.0% (145/966) of patients had PC (total incidence: 39.3%, 380/966). Among the 380 cases with PCs, the proportions of Clavien-Dindo grade Ⅰ, grade Ⅱ, grade Ⅲ and grade Ⅳ or above complications were 6.8%, 75.3%, 15.0% and 2.9%, respectively. Binary *Logistic* regressions showed that preoperative forced expiratory volume in one second (FEV_1_), diffusion capacity for carbon monoxide of the lung (single breath)(DLco SB), and preoperative combined chronic obstructive pulmonary disease were the significant independent factors for PCs. FEV_1_ in preoperative pulmonary function was the significant risk factor for complications at Clavien-Dindo grade Ⅰ, grade Ⅱ, grade Ⅲ, and above.

**Conclusion:**

Clavien-Dindo grade Ⅱ complications are the most common complications within 30 days after lung cancer lobectomy. FEV_1_ is closely related to the occurrence of PCs and may potentially be one of the practical variables to assess the risk of occurrence of PCs.

外科手术是早期肺癌的重要治疗措施，联合适当的辅助治疗可以给肺癌患者带来极大的获益。同时，手术也带来一些不可避免的副作用，例如与其他治疗选择的冲突、对术后生活质量的影响、治疗花费的增加、术后并发症及死亡的风险等^[1]^。其中术后并发症的发生引起胸外科领域医护人员极大的关注。

预防术后并发症成为肺癌手术治疗过程的重要问题之一。旨在有效降低术后并发症的策略如综合的术前评估、合理的术前心肺功能评定、优化的手术方式、标准化的护理监测等，近年得到较为广泛的认可和推广^[2-5]^。然而由于不同的医疗机构的技术水平、术后并发症的定义和评价标准的差异，与住院相关的术后并发症的评估分析也存在较大不同^[6-8]^，从而降低了不同医疗机构之间的数据、结论的可比性以及分析结果的一致性和推广性。因此，一套标准的、客观的并发症评价标准来更好地评价术后并发症，并探明这些并发症发生的危险因素，将有助于预防并发症的发生降低术后并发症的发生率、改善手术的效果。

1992年，Clavien等^[9]^根据术后并发症是否需要医疗干预建立了一套针对术后并发症的等级评价系统。系统制定了不同医疗机构之间、不同手术时期术后并发症的统一评价标准，极大地增加了资料间的可比性^[10]^。该并发症评价体系在2004年经Dindo等改进后，具有更普遍的适用性，并最终被命名为Clavien-Dindo并发症分级评价体系^[11-15]^。然而，应用该体系在肺癌手术领域进行并发症的分析研究相对较少^[16]^。基于此，我们使用Clavien-Dindo分级系统来分析肺癌患者肺叶切除术后短期并发症的发生情况并探寻不同并发症分级的危险因素。

## 1 资料与方法

研究调查了2013年6月-2014年12月间四川大学华西医院胸外科收治的966例行肺叶切除的肺癌患者。收集的信息包括患者年龄、吸烟史、术前合并症、术前肺功能、术前及术后血液检测指标（如白蛋白、白细胞计数等）、术后住院日和手术相关数据（如手术时间、手术方式、术中失血量等）。该研究是一项观察性和回顾性研究，已通过四川大学华西医院伦理委员会的审查。

### 1.1 纳入/排除标准

（1）纳入标准：①手术方式为肺叶切除术+系统淋巴结清扫术；②术后病理证实为非小细胞肺癌者。（2）排除标准：①肺部肿瘤为转移癌；②数据不全或相关资料缺失者。

### 1.2 Clavien-Dindo分级系统

Clavien-Dindo分级系统基于术后并发症的严重程度和是否需要针对并发症进行相应的治疗，将术后并发症分为Ⅰ级-Ⅴ级，Ⅰ级：术后出现的无需药物、手术、内镜或放射治疗的异常改变，但包括需要止吐药、解热药、止痛药、电解质和物理治疗的并发症，还包括需要在床旁行开放引流的伤口感染；Ⅱ级：需要除Ⅰ级所用药物以外的药物治疗，还包括输血和全胃肠外营养；Ⅲ级：需要行手术、内镜、放射治疗等干预措施，Ⅲa级：干预措施不需要在全麻下进行，Ⅲb级：干预措施需要在全麻下进行；Ⅳ级：危及生命的并发症，包括中枢神经系统并发症、需要重症监护或至重症监护病房处理的并发症，Ⅳa级：单个器官功能障碍（包括透析），Ⅳb级：多器官功能障碍；Ⅴ级：死亡^[17]^。

### 1.3 肺部感染诊断标准

肺部感染的诊断必须至少满足以下中的3条标准：①痰液或胸水中查见病原菌或支气管镜查见脓性分泌物；②胸部平片提示肺部渗出、实变影；③发热38 ℃以上；④白细胞>10, 000/mm^3^或 < 3, 000/mm^3^^[18]^。

### 1.4 统计学方法

数据采用SPSS 19.0（IBM Corp, Armonk, NY, USA）和Stata 12.0（Stata Corp, College station, TX）软件进行分析。计量资料采用均值±标准差（Mean±SD）表示，计数资料采用卡方检验或者*Fisher*确切概率法。并发症危险因素采用*Logistic*回归分析。单变量分析有统计意义的变量进行*Logistic*回归分析，预测术后并发症的独立危险因素。所有数据采用双侧检验，*P* < 0.05为差异有统计学意义。

## 2 结果

研究最终纳入966例患者，其中并发症组占15.0%（145/966）（表 1，表 2），并发症总次数380次。使用Clavien-Dindo分级系统将此380次并发症进行分级，Ⅰ级、Ⅱ级、Ⅲ级、Ⅳ级及以上并发症分别占6.8%、75.3%、15.0%和2.9%（表 3）。并发症发生率首位的是肺部感染（19.5%），其他主要并发症包括需要行加压引流的肺不张（8.4%）、≥7 d的肺漏气（7.4%）、胸腔积液（6.8%）、心率失常（5.8%）、肺水肿（5.5%）、切口感染（4.7%）以及需要行纤维支气管吸痰的痰潴留（4.7%）（表 3）。

**1:** 两组患者基线资料 Baseline characteristics

Characteristics	Non-PCs	PCs	*P*
Number	821 (85.0%)	145 (15.0%)	
Age (Mean±SD)	58.8±9.2	60.2±10.1	0.048
Gender			0.452
Men	569 (74.7%)	105 (72.4%)	
Female	252 (25.3%)	40 (27.6%)	
Smoking history			0.712
Never	286 (34.8%)	55 (37.9%)	
Current or former	535 (63.2%)	90 (62.1%)	
Preoperative FEV_1_(L)	2.5±0.18	2.07±0.22	0.001
Preoperative FVC (L)	3.2±0.84	3.27±0.76	0.771
Preoperative MVV (L/min)	96.7±24.1	96.4±26.4	0.821
Preoperative DLco (mL/min/mmHg)	24.1±4.7	20.3±3.9	< 0.001
Pathological type			< 0.001
Adenocarcinoma	576 (70.1%)	96 (66.2%)	
Squamous carcinoma	198 (24.1%)	40 (27.6%)	
Other	47 (5.8%)	9 (6.2%)	
Preoperative comorbidities			
COPD	22 (2.6%)	70 (48.3%)	< 0.001
Diabetes mellitus	35 (4.2%)	14 (9.7%)	0.006
Hypertension	22 (2.6%)	15 (10.3%)	< 0.001
Clinical stage			0.753
Stage Ⅰ or 0	535 (65.2%)	90 (62.1%)	
Stage Ⅱ	227 (27.6%)	41 (28.3%)	
Stage Ⅲ	51 (6.2%)	12 (8.3%)	
Stage Ⅳ	8 (1.0%)	2 (1.3%)	
SD: standard deviation; FEV_1_: forced expiratory volume in one second; FVC: forced vital capacity; MVV: maximum ventilatory volume; DLco: diffusion capacity for carbon monoxide of the lung; COPD: chronic obstructive pulmonary disease.			

**2:** 两组患者临床特征 Clinical characteristics between the two groups

Characteristics	Non-PCs	PCs	*P*
Surgical approach			0.012
VATS	440 (53.6%)	51 (35.2%)	
Open	381 (43.4%)	94 (64.8%)	
WBC count(×10^9^/L)			
Preoperative	7.0±1.7	7.8±1.9	0.041
Postoperative	16.5±3.8	17.8±3.5	< 0.001
Albumin (g/L)			
Preoperative	43.0±1.8	42.0±2.2	< 0.001
Postoperative	30.4±3.1	30.0±3.3	0.192
Operation time (min)	116.7±31.7	135.0±2.2	< 0.001
Amount of intraoperative bleeding (mL)	179.5±46.7	178.9±42.7	0.890
Average time with the drainage tube (d)	7.0±1.8	10.2±2.3	< 0.001
Average time of in-hospital stay	13.6±5.8	14.9±5.5	0.015
In-hospital expense (¥)	30, 644.5±4, 784.3	40, 999.9±2, 396.0	< 0.001
Antibiotic use			
Average time of use (d)	6.9±1.2	9.8±2.3	< 0.001
Second-generation cephalosporins	796 (96.9%)	105 (72.4%)	< 0.001
Third-generation or other	25 (3.1%)	40 (27.6%)	< 0.001
VATS: video-assisted thoracic surgery; WBC: white blood cell.			

**3:** Clavien-Dindo分级系统对肺癌患者术后并发症分级 Complications classified by the Clavien-Dindo Classification of Surgical Complications

Grade	Occurring time	Percentage (%)
Grade Ⅰ		
Aerothorax ( < 3 d)	8	2.1
Persistent pulmonary leakage ( < 7 d)	12	3.2
Others	6	1.6
Grade Ⅱ		
Persistent pulmonary leakage (≥7 d)	28	7.4
Aerodermectasia	46	12.1
Wound infection	18	4.7
Arrhythmia	22	5.8
Pneumonedema	21	5.5
Increased blood pressure	27	7.1
Atelectasis needing suction	32	8.4
Pneumonia	74	19.5
Thrombus of lower limb	7	1.8
Urinary tract infection	3	0.8
Phrenitis	1	0.3
Others	7	1.8
Grade Ⅲ		
Pleural effusion needing intervention	26	6.8
Persistent atelectasis needing intervention (Sputum aspiration, *e.g*.)	18	4.7
Aerodermectasia	6	1.6
Chylopleura	2	0.5
Postoperative pleural hemorrhage needing surgery	3	0.8
Bronchopleural fistula	2	0.5
Above grade Ⅲ		
Respiratory failure	2	0.5
Return to ICU	6	1.6
Other like severe pneumonia resulting in organ failure or death	3	0.8
ICU: intensive care unit.		

并发症组的平均年龄明显高于无并发症组[(60.2±0.1) yr *vs* (58.8±9.2) yr, *P*=0.048]。并发症组术前合并高血压（10.3%, 15/145 *vs* 2.6%, 22/821, *P* < 0.001）、慢性阻塞性肺疾病（chronic obstructive pulmonary disease, COPD）（48.3%, 70/145, *vs* 2.6%, 22/821, *P* < 0.001）、糖尿病（9.7%, 14/145 *vs* 4.2%, 35/821, *P*=0.006）的比例都明显高于无并发症组，并发症组术前肺一氧化碳弥散量（diffusion capacity for carbon monoxide of the lung single breath, DLco SB）[(20.3±3.9) mL/min/mmHg *vs* (24.1±4.7) mL/min/mmHg, *P* < 0.001]明显低于无并发症组。并发症组在总住院时间[(14.9±5.5) d *vs* (13.6±5.8) d, *P*=0.015]、手术时间[(135.0±2.2) min *vs* (116.7±31.7) min, *P* < 0.001]、术后带管时间[(10.2±2.3) d *vs* (7.0±1.8) d, *P* < 0.001]、住院总花费[¥(40, 999.9±2, 396.0) *vs* ¥(30, 644.5±4, 784.3), *P* < 0.001]、抗生素使用时间[(9.8±2.3) d *vs* (6.9±1.2) d, *P* < 0.001]、术前白细胞计数[(7.8±1.9)×10^9^ *vs* (7.0±1.7)×10^9^, *P*=0.041]、术后白细胞计数[(17.8±3.5)×10^9^ *vs* (16.5±3.8)×10^9^, *P* < 0.001]都明显高于无并发症组，并发症组术前血清白蛋白含量[(42.0±2.2) *vs* (43.0±1.8), *P* < 0.001]明显低于无并发症组，两组患者的病理类型比例存在明显差异（*P* < 0.001），并发症组鳞癌比例较高（40/145, 27.6% *vs* 198/821, 24.1%），而无并发症组腺癌比例较高（576/821, 70.1% *vs* 96/145, 66.2%）（表 1）。

*Logistic*回归分析结果显示发生术后并发症的危险因素包括第1秒用力呼气容积（forced expiratory volume in one second, FEV_1_）（OR=2.322, 95%CI: 1.373-1.921, *P*=0.004）、DLco SB（OR=2.137, 95%CI: 1.298-1.873, *P*=0.007）和术前合并COPD（OR=1.763, 95%CI: 1.189-1.684, *P*=0.028）（表 4）。

**4:** 对临床变量和术后并发症的关系进行多变量分析 Multivariate analysis of the association between clinical variables and PCs

	*P*	Exp(B)	95%CI	
			Lower	Upper
Age	0.126	1.03	1.004	1.058
Preoperative FEV_1_	0.004	2.322	1.373	1.921
Preoperative albumin	0.071	0.74	0.648	0.846
Preoperative WBC count	0.067	0.771	0.67	0.887
Operation time	0.102	1.024	1.015	1.033
Preoperative hypertension	0.143	1.182	0.768	1.194
Preoperative diabetes mellitus	0.067	0.275	0.069	1.095
Preoperative COPD	0.028	1.763	1.189	1.684
VATS	0.066	1.623	0.968	2.721
Dlco SB	0.007	2.137	1.298	1.873
Constant	0.049	0.001		

术前FEV_1_是Clavien-Dindo Ⅰ级（OR=1.469, 95%CI: 1.488-1.891, *P*=0.015）、Ⅱ级（OR=1.657, 95%CI: 1.069-1.515, *P*=0.003）、Ⅲ级及以上（OR=1.241, 95%CI: 1.137-1.652, *P*=0.023）并发症的独立危险因素（表 5）。

**5:** 对主要临床变量和术后并发症分级的关系进行多变量分析 Multivariate analysis of the association between main clinical variables and grades of PCs

	*P*	Exp(B)	95%CI	
			Lower	Upper
Grade Ⅰ				
Preoperative FEV_1_	0.015	1.469	1.488	1.891
Operation time	0.002	1.016	1.006	1.027
Preoperative COPD	0.000	8.696	1.638	1.783
Grade Ⅱ				
Preoperative FEV_1_	0.003	1.657	1.069	1.515
Operation time	0.017	1.024	1.015	1.032
DLco SB	0.001	2.112	1.201	1.799
Grade Ⅲ or above				
Preoperative FEV_1_	0.023	1.241	1.137	1.652
We listed the variables with statistical significance (*P* < 0.05) analyzed via *Logistic* analysis.				

## 3 讨论

对手术患者进行客观、有效的手术风险评估对临床医生和患者都有极大的获益。然而，由于并发症的复杂性和多样性使临床上评估工作的实施面临诸多困难。一个标准的、具有可操作性的、综合的术后并发症评价系统有助于临床医生制定相应诊疗策略，并降低不同医疗机构的差异引起的偏倚，最终指导改善患者的预后。

针对术后并发症的Clavien-Dindo分级系统在其他外科领域的应用已有较多报道，而用于评价肺癌患者肺叶切除术后并发症，尚属少见。本研究中，我们纳入肺叶切除的患者，避免由于手术方式例如肺楔形切除、肺段切除或全肺切除引起的偏倚；同时，对手术并发症进行分级，并针对不同的并发症分级进行统计分析；另外，为降低人工偏倚的影响，确保数据的真实、有效性，我们全面查阅了电子版和纸质版医疗记录。最终研究纳入966例肺癌患者，其中并发症组145例（15.0%），此并发症发生率与其他报道较为一致^[19-23]^。并发症组患者的平均年龄明显高于无并发症组，但危险因素分析结果显示，年龄不是术后并发症的独立危险因素。而年龄与术后并发症的关系尚存争议，一项包含727例患者的研究结果^[24]^也显示年龄与术后并发症的发生率没有特定的联系；但也有研究^[25]^认为年龄>70岁是肺癌患者术后并发症的危险因素之一。因此，仍需更加深入研究年龄和术后并发症发生的联系。此前的一项研究^[26]^显示吸烟和不良预后之间没有关系，我们的研究结果也显示吸烟不是术后并发症的危险因素，但值得注意的是，长期吸烟对肺功能有较大的影响，而肺功能与术后并发症的发生率和严重程度密切相关。因此，吸烟与术后并发症的关系不能忽视，仍需后续深入的研究加以证实。

本研究结果显示并发症组合并COPD、糖尿病和高血压比例明显高于无并发症组。此前的一项研究^[27]^发现术前合并症与发生术后并发症相关。同时，合并COPD是发生术后并发症的独立危险因素^[28]^。尽管高血压被认为不是术后并发症的危险因素，但高血压与心血管系统有密切联系，同时心、脑血管事件在高血压患者更常见，因此，高血压对手术预后的影响依旧不可忽视^[29]^。在我们的研究中，术后平均住院日、手术时间、术后带管时间、住院花费和抗生素使用时间在并发症组明显高于无并发症组，这表明术后并发症不仅影响患者预后和生活质量，也增加了相应的医疗费用。抗生素广泛应用于外科手术患者的围术期管理，尤其是存在术后感染风险的患者^[30-32]^。然而，目前术后抗生素的使用并没有相应的指南，多数医疗机构普遍使用一代或二代头孢类抗生素来预防术后感染^[33]^。本研究发现并发症组抗生素的使用时间更长且使用的抗生素级别更高，也从侧面说明并发症组更长的住院时间以及更高的住院花费。电视辅助胸腔镜手术（video-assisted thoracoscopic surgery, VATS）能减轻术后疼痛、减少引流量、缩短手术时间、缩短住院日、与辅助治疗能更好地结合，是早期非小细胞肺癌广泛使用的手术方式^[34, 35]^。美国国家综合癌症网络（National Comprehensive Cancer Network, NCCN）指南同样建议VATS为治疗早期非小细胞肺癌的首选方式。无并发症组较高的VATS使用比例同样也反映了其在肺癌外科治疗方面的优势。

在并发症的Clavien-Dindo分级中，Ⅱ级并发症的比例最高。发生率较高的主要并发症包括肺部感染、皮下气肿、胸腔积气、需要吸痰的肺不张、持续性肺漏气、心率失常、肺水肿、切口感染等。术前FEV_1_的平均值在无并发症组明显高于并发症组，进一步多变量分析结果显示术前FEV_1_是术后Clavien-Dindo Ⅰ级、Ⅱ级、Ⅲ级、Ⅳ级及以上并发症的独立危险因素，验证了FEV_1_与术后并发症的紧密联系。据报道^[36, 37]^，与FEV_1_%>70%的患者相比，FEV_1_% < 70%的患者术后并发症的发生率更高，表明术前肺功能对预测术后并发症有重要意义。因此，近年大量研究建议对肺功能有损伤的患者行围术期物理康复治疗、应用皮质类固醇和支气管扩张剂、严格戒烟，以有效减少肺功能损伤，并最终降低患者术后并发症的发生率^[38]^。

在本研究中我们使用了Clavien-Dindo分级系统来评价肺癌患者肺叶切除术的术后并发症，有助于进行科学的研究并为临床决策提供参考。例如，基于是否需要医疗干预，持续性肺漏气被精确地分为Clavien-Dindo Ⅰ级或Ⅱ级。而其他大多数研究根据术后是否出现肺不张进行分析，没有根据Clavien-Dindo分级进行研究（比如Clavien-Dindo Ⅱ级的肺不张需要吸痰，而Ⅲ级肺不张需要行纤维支气管镜吸痰）。同时本研究也存在一些不足。首先，这是一项回顾性研究，患者均来自单个医疗中心的手术患者，降低了此分级系统在其他医疗中心应用的推广性。其次，没有分析患者术后长期生活质量或长期并发症发病率等，也没有分析术后第1秒用力呼气容积的预测值（ppoFEV_1_）对并发症发生率的影响，降低了综合评价的有效性。因此，需要更大样本含量的前瞻性研究深入探讨Clavien-Dindo分级系统下，肺癌术后并发症的相关问题。

Clavien-Dindo分级系统下，Clavien-Dindo Ⅱ级的并发症在肺叶切除术后最常见，而FEV_1_与肺癌患者术后并发症的发生密切相关，是评估术后并发症发生风险的可靠指标之一。

## References

[1] Roeslin N, Morand G (1992). Complications and mortality of surgery for bronchogenic cancers. Rev Pneumol Clin.

[2] Kaza AK, Mitchell JD (2005). Preoperative pulmonary evaluation of the thoracic surgical patient. Thorac Surg Clin.

[3] Doyle RL (1999). Assessing and modifying the risk of postoperative pulmonary complications. Chest.

[4] Wilson DJ (1997). Pulmonary rehabilitation exercise program for high-risk thoracic surgical patients. Chest Surg Clin N Am.

[5] Eric MT, David HH (2002). Operative risk in thoracic surgery. Chest Surg Clin N Am.

[6] Shiono S, Yoshida J, Nishimura M (2007). Risk factors of postoperative respiratory infections in lung cancer surgery. J Thorac Oncol.

[7] Takenaka T, Katsura M, Shikada Y (2013). The impact of cardiovascular comorbidities on the outcome of surgery for non-small-cell lung cancer. Interact Cardiovasc Thorac Surg.

[8] Rueth NM, Parsons HM, Habermann EB (2012). Surgical treatment of lung cancer: predicting postoperative morbidity in the elderly population. J Thorac Cardiovasc Surg.

[9] Clavien PA, Sanabria JR, Strasberg SM (1992). Proposed classification of complications of surgery with examples of utility in cholecystectomy. Surgery.

[10] Dindo D, Demartines N, Clavien PA (2004). Classification of surgical complications: A new proposal with evaluation in a cohort of 6, 336 patients and results of a survey. Ann Surg.

[11] Gonzalgo ML, Pavlovich CP, Trock BJ (2005). Classification and trends of perioperative morbidities following laparoscopic radical prostatectomy. J Urol.

[12] Kocak B, Koffron AJ, Baker TB (2006). Proposed classification of complications after live donor nephrectomy. Urology.

[13] Tamura S, Sugawara Y, Kaneko J (2006). Systematic grading of surgical complications in live liver donors according to Clavien's system. Transpl Int.

[14] DeOliveira ML, Winter JM, Schafer M (2006). Assessment of complications after pancreatic surgery: a novel grading system applied to 633 patients undergoing pancreaticoduodenectomy. Ann Surg.

[15] Seely AJ, Ivanovic J, Threader J (2010). Systematic classification of morbidity and mortality after thoracic surgery. Ann Thorac Surg.

[16] Amar D, Munoz D, Shi W (2010). A clinical prediction rule for pulmonary complications after thoracic surgery for primary lung cancer. Anesth Analg.

[17] Zeng K, Wang QZ, Li YL (2016). Application of Clavien-Dindo Classification System to complications of minimally-invasive percutaneous nephrolithotomy. Zhongguo Xian Dai Yi Xue Za Zhi.

[18] Lai YT, Su JH, Yang M (2016). Impact and effect of preoperative short-term pulmonary rehabilitation training on lung cancer patients with mild to moderate chronic obstructive pulmonary disease: a randomized trial. Zhongguo Fei Ai Za Zhi.

[19] Sabaté S, Mazo V, Canet J (2014). Predicting postoperative pulmonary complications: implications for outcomes and costs. Curr Opin Anaesthesiol.

[20] Langeron O, Carreira S, le Saché F (2014). Postoperative pulmonary complications updating. Ann Fr Anesth Reanim.

[21] Stiles BM, Poon A, Giambrone GP (2016). Incidence and factors associated with hospital readmission after pulmonary lobectomy. Ann Thorac Surg.

[22] Madani A, Jr Fiore JF, Wang Y (2015). An enhanced recovery pathway reduces duration of stay and complications after open pulmonary lobectomy. Surgery.

[23] Brunelli A, Refai M, Xiumé F (2008). Performance at symptom-limited stair-climbing test is associated with increased cardiopulmonary complications, mortality, and costs after major lung resection. Ann Thorac Surg.

[24] Ogawa F, Wang G, Matsui Y (2013). Risk factors for postoperative complications in the elderly with lung cancer. Asian Cardiovasc Thorac Ann.

[25] Wang Z, Zhang J, Cheng Z (2014). Factors affecting major morbidity after video-assisted thoracic surgery for lung cancer. J Surg Res.

[26] Shiono S, Katahira M, Abiko M (2015). Smoking is a perioperative risk factor and prognostic factor for lung cancer surgery. Gen Thorac Cardiovasc Surg.

[27] Sekine Y, Yamada Y, Chiyo M (2007). Association of chronic obstructive pulmonary disease and tumor recurrence in patients with stage IA lung cancer after complete resection. Ann Thorac Surg.

[28] Sekine Y, Behnia M, Fujisawa T (2002). Impact of COPD on pulmonary complications and on long-term survival of patients undergoing surgery for NSCLC. Lung Cancer.

[29] Bloomfield GS, Wang TY, Boulware LE (2015). Implementation of management strategies for diabetes and hypertension: from local to global health in cardiovascular diseases. Glob Heart.

[30] Jones DJ, Bunn F, Bell-Syer SV (2014). Prophylactic antibiotics to prevent surgical site infection after breast cancer surgery. Cochrane Database Syst Rev.

[31] Gower EW, Lindsley K, Nanji AA (2013). Perioperative antibiotics for prevention of acute endophthalmitis after cataract surgery. Cochrane Database Syst Rev.

[32] Brignardello-Petersen R, Carrasco-Labra A (2014). Antibiotic prophylaxis for preventing infectious complications in orthognathic surgery. Cochrane Database Syst Rev.

[33] Edwards FH, Engelman RM, Houck P (2006). The Society of Thoracic Surgeons practice guideline series: antibiotic prophylaxis in cardiac surgery. Part Ⅰ: duration. Ann Thorac Surg.

[34] Morikawa T (2006). Thoracoscopic surgery for lung cancer. Ann Thorac Cardiovasc Surg.

[35] Takei H, Goya T, Tsukada H (2005). Video-assisted thoracic surgery (VATS) for lung cancer. Gan To Kagaku Ryoho.

[36] Win T, Jackson A, Sharples L (2005). Relationship between pulmonary function and lung cancer surgical outcome. Eur Respir J.

[37] Powell CA, Caplan CE (2001). Pulmonary function tests in preoperative pulmonary evaluation. Clin Chest Med.

[38] Wong DH, Weber EC, Schell MJ (1995). Factors associated with postoperative pulmonary complications in patients with severe chronic obstructive pulmonary disease. Anesth Analg.

